# Optofluidic Lens Refractometer

**DOI:** 10.3390/mi16101160

**Published:** 2025-10-13

**Authors:** Yifan Zhang, Qi Wang, Yuxiang Li, Junjie Liu, Ziyue Lin, Mingkai Fan, Yichi Zhang, Xiang Wu

**Affiliations:** The Key Laboratory of Micro and Nano Photonic Structures, Department of Optical Science and Engineering, Fudan University, Shanghai 200438, China; 23210720014@m.fudan.edu.cn (Y.Z.); 22110720127@m.fudan.edu.cn (Q.W.); 21110720012@m.fudan.edu.cn (Y.L.); 22201720011@m.fudan.edu.cn (J.L.); 23210720102@m.fudan.edu.cn (Z.L.); 23110720004@m.fudan.edu.cn (M.F.); yichizhang24@m.fudan.edu.cn (Y.Z.)

**Keywords:** microlens, optofluidic, refractometer, sensors, gradient boosting decision tree, image-based signal processing, beam transformation

## Abstract

In the face of increasingly severe global environmental challenges, the development of low-cost, high-precision, and easily integrable environmental monitoring sensors is of paramount importance. Existing optical refractive index sensors are often limited in application due to their complex structures and high costs, or their bulky size and difficulty in automation. This paper proposes a novel optical microfluidic refractometer, consisting solely of a laser source, an optical microfluidic lens, and a CCD detector. Through an innovative “simple structure + algorithm” design, the sensor achieves high-precision measurement while significantly reducing cost and size and enhancing robustness. With the aid of signal processing algorithms, the device currently enables the detection of refractive index gradients as low as 1.4 × 10^−5^ within a refractive index range of 1.33 to 1.48.

## 1. Introduction

Currently, the world is facing unprecedented environmental challenges, including climate change, widespread pollution, and ecosystem degradation, which profoundly impact both natural stability and human societies [1,2]. This situation creates an urgent need for precise, efficient, and real-time monitoring technologies. Among the potential solutions, optical refractive index sensors are emerging as a particularly promising technology, owing to their unique advantages such as high sensitivity, rapid response, non-invasive measurement, resistance to electromagnetic interference, as well as ease of integration and miniaturization [3,4,5,6]. They have been widely applied to the real-time monitoring of water quality and pollutant concentrations, providing an indispensable data foundation for environmental protection decision-making and pollution control [7].

However, traditional optical refractive index sensors still face numerous challenges in practical large-scale applications. Mainstream optical refractive index sensors can generally be classified into resonance-based and refraction-based types. Resonance-based sensors, such as those relying on on-chip periodic nanostructures (e.g., photonic crystals [8], metasurfaces [9]), optical microcavities (e.g., microring resonators [10], Fabry–Pérot cavities [11]), and surface plasmon resonance (SPR) devices [12], can achieve extremely high detection sensitivity. Nevertheless, their performance is highly dependent on precise micro/nanofabrication processes and specific material selections. This not only results in high manufacturing costs, complex fabrication processes, and difficulties in controlling yield and ensuring robustness, but also poses challenges for practical commercialization [13,14]. Moreover, in order to excite and precisely detect sharp resonance peaks to obtain high-accuracy data, resonant sensors often require wavelength-stabilized and highly monochromatic laser sources, high-sensitivity photodetector arrays, and data acquisition and signal processing units [15]. These requirements further increase the overall cost, size, and power consumption of the system, greatly limiting their prospects for application in scenarios requiring large-scale distributed deployment.

On the other hand, traditional refractive sensors, as represented by the classic Abbe refractometer, also face challenges. High-precision laboratory Abbe refractometers are typically bulky and expensive, making them difficult to portabilize and integrate for in-situ applications. While portable models are more convenient, they often have relatively lower accuracy [16]. Furthermore, their inherent lack of microfluidic channels hinders their ability to perform continuous, real-time measurements, thereby limiting their application in dynamic and complex environmental monitoring scenarios. Other refraction-type refractive index sensors are relatively few, primarily involving two approaches: measuring the refractive index by observing image changes after a light beam passes through a test lens or transparent solid [17,18,19]; or correcting the light spot passing through the test liquid using a voltage-adjustable lens in a liquid cell and inferring refractive index changes based on voltage variations [20]. However, these methods struggle to achieve real-time measurements of liquids and suffer from low precision due to simplistic image processing techniques. While integrating microfluidic channels holds potential for addressing these issues, existing solutions [21,22] exhibit relatively low detection accuracy, failing to support precise measurements. To date, achieving high-precision measurements using refractive effects necessitates combining with other effects [23,24,25], making it challenging to overcome the issue of high costs through a simplistic refraction principle alone.

**Figure 1:**
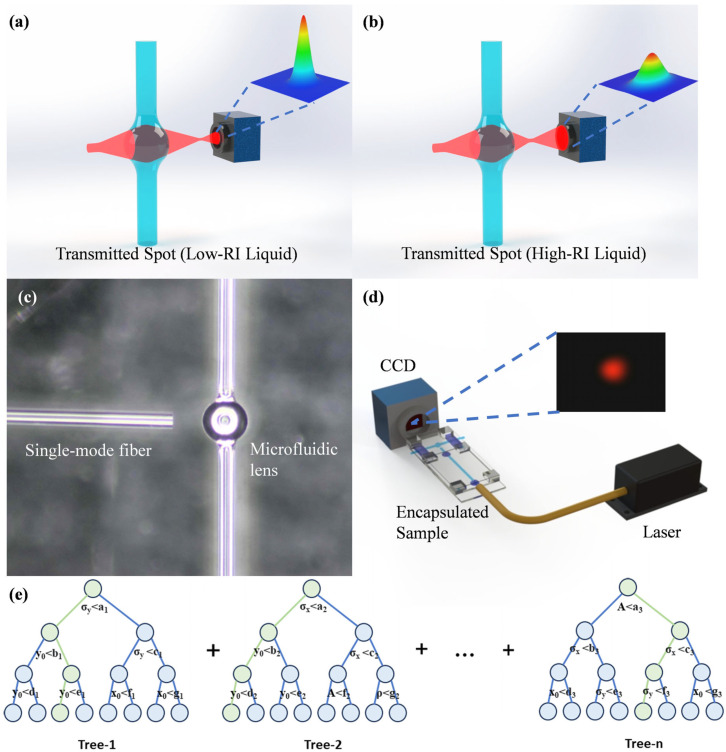
(**a**) A Gaussian beam passing through a low-refractive-index liquid. (**b**) A Gaussian beam passing through a high-refractive-index liquid. (**c**) Magnified microscopic image of the microbubble region. (**d**) Schematic of the optical path. (**e**) Schematic diagram of the Gradient Boosting Decision Tree (the green area illustrates a single decision iteration).

To address the high cost, large size, limited robustness, and challenges in continuous measurement faced by traditional refractive index sensors in environmental monitoring, as shown in Table 1, we have designed a novel optofluidic microlens refractometer. This device is engineered for the low refractive index measurement of liquids, enabling real-time monitoring through a computer-interfaced microfluidic channel. As shown in Figure 1d, its core structure is extremely simplified, consisting of only a low-cost semiconductor laser source, an optofluidic microlens, and a CCD image sensor. The principle is straightforward: as the Gaussian beam passes through the microlens, a lower refractive index results in a smaller focal spot, whereas a higher refractive index leads to a larger spot, as illustrated in Figure 1a,b. Therefore, variations in spot size can indicate changes in the refractive index of the liquid. As shown in Figure 1c, the device is highly compact. This design not only significantly reduces manufacturing costs and physical size but also features strong robustness to fabrication errors, making it suitable for practical applications by leveraging a “minimalist structure + algorithm” approach: image processing algorithms are used to fit and analyze data from the CCD, and gradient boosting decision tree algorithms [26,27,28] are employed to further suppress data noise. As a result, the system achieves measurement accuracy and sensitivity comparable to—or even surpassing—those of traditional, complex sensors, all while dramatically simplifying the structural complexity. This strategy of enhancing measurement precision through algorithms and improving robustness through structural simplification offers an innovative path forward for the current challenges in sensor development.

This paper will systematically elaborate on the physical structure and working principle of the optofluidic microlens refractometer, and demonstrate its key performance metrics through experimental data. The aim is to provide a new-generation sensing technology solution for the field of environmental monitoring—one that is more cost-effective, offers higher reliability, and is better suited for real-time measurement.

## 2. Theoretical Analysis and Numerical Simulation

### 2.1. ABCD Matrix

**Figure 2:**
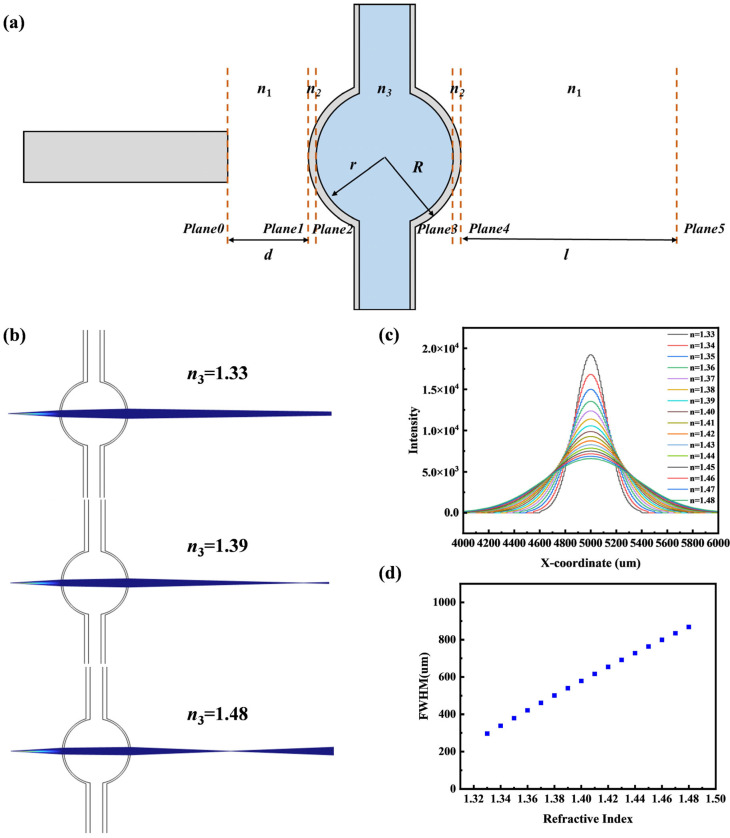
(**a**) ABCD matrix model. (**b**) Gaussian beams in the liquid core for different refractive indices. (**c**) 1D light intensity distributions for different refractive indices. (**d**) Relationship between refractive index and FWHM.

To validate the feasibility of inferring the liquid’s refractive index from the resulting beam spot, we constructed the ABCD matrix for the optical path from the single-mode fiber through the optofluidic lens to the CCD, as shown in Figure 2a. The overall system can be expressed as follows [29,30]:

(1) $$M_{01}=\left[\begin{array}{cc} 1 & d\\ 0 & 1 \end{array}\right],\;M_{1}=\left[\begin{array}{cc} 1 & 0\\ \frac{n_{1}-n_{2}}{n_{2}R} & \frac{n_{1}}{n_{2}} \end{array}\right],\;{\;M}_{12}=\left[\begin{array}{cc} 1 & R-r\\ 0 & 1 \end{array}\right],\;M_{2}=\left[\begin{array}{cc} 1 & 0\\ \frac{n_{2}-n_{3}}{n_{3}r} & \frac{n_{2}}{n_{3}} \end{array}\right]\;M_{23}=\left[\begin{array}{cc} 1 & 2r\\ 0 & 1 \end{array}\right],\;M_{3}=\left[\begin{array}{cc} 1 & 0\\ -\frac{n_{3}-n_{2}}{n_{2}r} & \frac{n_{3}}{n_{2}} \end{array}\right],\;M_{34}=\left[\begin{array}{cc} 1 & R-r\\ 0 & 1 \end{array}\right],\;M_{4}=\left[\begin{array}{cc} 1 & 0\\ -\frac{n_{2}-n_{1}}{n_{1}R} & \frac{n_{2}}{n_{1}} \end{array}\right],\;\;M_{45}=\left[\begin{array}{cc} 1 & l\\ 0 & 1 \end{array}\right],$$

where $n_{1}$ is the refractive index of air, $n_{2}$ and $n_{3}$ are the refractive indices of the microbubble wall and the liquid inside the microbubble. d denotes the distance from the single-mode fiber to the microbubble wall, while *r* and *R* represent the inner and outer radii of the microbubble, respectively. l is the distance between the microbubble and the CCD. Therefore, the overall ABCD matrix of the system can be expressed as:

(2) $$M=M_{45}\cdot M_{4}\cdot M_{34}\cdot M_{3}\cdot M_{23}\cdot M_{2}\cdot M_{12}\cdot M_{1}\cdot M_{01},$$

When the Gaussian beam propagates from surface 0 to surface 5, it can be calculated using the following formula:

(3) $$q\left(z_{5}\right)=\frac{A\cdot q\left(z_{0}\right)+B}{C\cdot q\left(z_{0}\right)+D},$$

where q(z) is a parameter associated with the Gaussian beam characteristics:

(4) $$\frac{1}{q(z)}=\frac{1}{R(z)}-i\cdot \frac{\lambda }{\pi \omega (z{)}^{2}n},$$

where R(z) is the wavefront radius of curvature, λ is the central wavelength, ω(z) is the beam waist radius, and n is the effective refractive index. By solving the equation, the Gaussian beam parameters on the CCD can be calculated.

### 2.2. Numerical Simulation

In this study, the Geometrical Optics Module within the finite element software COMSOL Multiphysics 6.3 was employed to investigate the influence of various parameter variations on the performance of the optofluidic lens refractometer. This model computes ray trajectories, applying Snell’s law for refraction as the boundary condition at material interfaces; reflections were disregarded. The light source was modeled as a Gaussian beam with a wavelength of 660 nm and a beam waist radius of 2 µm. Furthermore, since the experimental setup utilizes a broadband light source, interference effects are considered negligible and are thus not captured by the geometrical optics model. For the initial simulation, the following parameters, corresponding to the schematic in Figure 2a, were fixed: the microbubble radius was set to 200 µm, the distance between the Gaussian light source and the optofluidic lens was 300 µm, the refractive index of the liquid core was 1.33, and the microbubble wall had a refractive index of 1.45 with a thickness of 1 µm. The distance from the microbubble to the CCD receiving surface was set to 3 cm.

The first part of the study investigates the effect of the liquid core refractive index on the beam spot. Under the initial parameters, the refractive index was gradually increased from 1.33 to 1.48. As shown in Figure 2b, with an increase in the liquid core refractive index, the focusing ability becomes stronger, and the focal point shifts toward the microbubble. Meanwhile, the beam spot on the CCD becomes more divergent as the refractive index increases. Figure 2c presents the variation in the intensity distribution of the beam spot corresponding to the change in refractive index, demonstrating the feasibility of this sensing approach. Furthermore, as illustrated in Figure 2d, the full width at half maximum (FWHM) increases with the refractive index.

### 2.3. Robustness Testing

#### 2.3.1. Microbubble Radius

**Figure 3:**
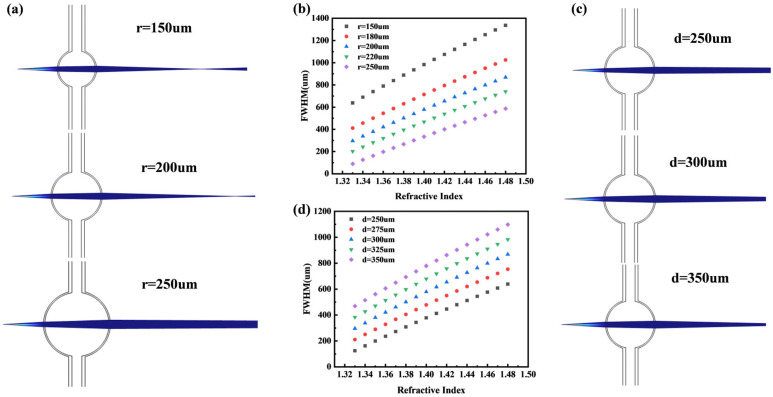
(**a**) Gaussian beams for different radii. (**b**) Variation of FWHM with refractive index for different radii. (**c**) Gaussian beams for different source spacings. (**d**) Variation of FWHM with refractive index for different source spacings.

Based on the initial parameters, we gradually increased the radius of the microbubble lens from 150 μm to 250 μm. As shown in Figure 3a, the focusing ability weakens with increasing radius, causing the focal point to shift toward the CCD and resulting in a smaller spot size. As illustrated in Figure 3b, for different microbubble radii, the refractive index maintains a good correspondence with the FWHM, indicating a consistent relationship across varying geometries.

#### 2.3.2. Distance from Light Source

As shown in Figure 3c, the distance between the microbubble and the light source was gradually increased from 250 μm to 350 μm. With increasing distance, the focal point shifts toward the microbubble, leading to an enlarged spot size on the CCD. As illustrated in Figure 3d, the FWHM still maintains a good correlation with the refractive index under different source distances, demonstrating the robustness of this relationship.

#### 2.3.3. Microbubble Height Offset

**Figure 4:**
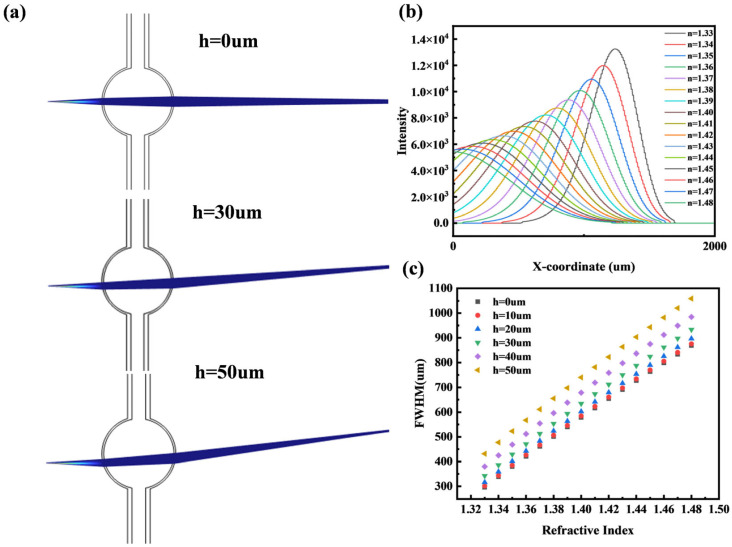
(**a**) Gaussian beams for different height offsets. (**b**) Light intensity distribution at a height offset of 50 µm. (**c**) Variation of FWHM with refractive index for different height offsets.

As shown in Figure 4a, we varied the vertical offset of the microbubble relative to the center of the light source from 0 to 50 μm. It can be observed that the spot displacement increases with the offset. Furthermore, as illustrated in Figure 4b, the spot displacement also increases with the refractive index, providing an additional dimensional parameter for refractive index measurement. As shown in Figure 4c, even with a vertical offset in the range of 0–50 μm, the refractive index still maintains a consistent correlation with the FWHM.

Through simulations involving variations in microbubble radius, light source distance, and vertical offset, we demonstrate that the proposed optofluidic bubble lens refractometer exhibits strong robustness against common fabrication imprecisions, confirming its wide operational range and practical viability.

## 3. Materials and Methods

### 3.1. Materials

The microlens is fabricated using a fused silica capillary tube, into which gas is introduced during the manufacturing process. In the first step, one end of the silica tube is sealed by melting it with an electric discharge, ensuring the internal cavity is closed. In the second step, the area designated for bubble formation is quickly heated using an alcohol lamp to carbonize any coating layer, which is then cleaned with alcohol to obtain a clean section of the silica tube. In the third step, the open end of the silica tube is connected to a syringe, allowing gas to be injected into the tube by pushing the syringe. In the fourth step, discharge heating is applied to the blown part using a relatively small discharge current, while simultaneously injecting gas internally. The size of the microbubbles can be controlled by adjusting the amount of injected gas. Repeating this process yields a microbubble structure.

The next stage involves sample preparation. First, a glass base is prepared. In the second step, a single-mode optical fiber (Model: DH-FSM600-APC-1, Daheng Optics, Beijing, China), which is single-mode for the 600–770 nm wavelength range and has a core diameter of 4.0 µm, is placed on the glass base. The end-face of the fiber was prepared by direct cleaving with a fiber cleaver, without subsequent polishing, and was then fixed in position using a UV-curable adhesive. In the third step, the microbubble is positioned appropriately using a five-axis translation stage and fixed to a glass holder with UV adhesive. In the final step, a coverslip is placed above the assembly and secured with UV adhesive, completing the fabrication of the sample. Magnified images and a schematic of the complete sample are shown in Figure 1c,d.

### 3.2. Method

To perform the measurements, a simple optical system was constructed. It utilizes a semiconductor laser (Model: FC-660-050-PM, 655–665 nm, Shanghai SFOLT Co., Ltd., Shanghai, China) to launch a Gaussian beam from a single-mode fiber through the optofluidic lens sensing device. As depicted in Figure 1d, the beam is ultimately imaged onto a CCD camera (Model: bc4800, Dongguan Bosheng Electronics, Dongguan, China) equipped with a sensor of approximately 6.08 mm × 4.56 mm, capturing images at a 1280 × 960-pixel resolution. The refractive index of the internal liquid is subsequently inferred by analyzing the captured CCD image. The total cost of the current system is approximately $1000, with the main cost concentrated on the laser. The sample cost can be controlled to within $5. A laser with poorer monochromaticity and a CCD with lower resolution could be used to optimize the cost.

## 4. Results and Discussion

### 4.1. Fitted Half-Width

**Figure 5:**
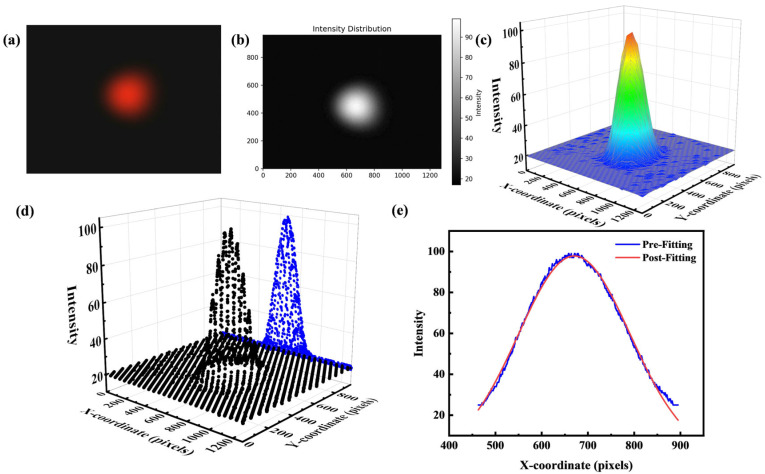
(**a**) Original spot image. (**b**) Grayscale image. (**c**) Intensity map. (**d**) Extracted 1D data: original intensity (black points) and its xz-plane projection (blue points). (**e**) 1D Gaussian fit.

For the raw CCD image shown in Figure 5a, we first converted it to a grayscale image, as shown in Figure 5b, to obtain the intensity information for each pixel, illustrated in Figure 5c. To characterize the intensity distribution of the beam spot along the x-direction, we extracted an intensity profile along the horizontal axis (x = a) of the image. Specifically, for each column of pixels in the image, we calculated the maximum intensity value, thereby obtaining intensity data as a function of x-coordinate, similar to the projection operation shown in Figure 5d. Next, we performed a Gaussian fitting on the intensity values that exceed a defined threshold, as shown in Figure 5e.

(5) $$G\left(x\right)=A\cdot \exp\left(-\frac{(x-\mu {)}^{2}}{2\sigma^{2}}\right),$$

In the Gaussian fitting, *A* represents the peak intensity (amplitude), *μ* denotes the x-coordinate of the beam center (mean), and *σ* corresponds to the standard deviation, which reflects the beam spot size. Based on the fitted parameters, the FWHM can be calculated using the formula:

(6) $$FWHM=2\sigma \sqrt{2ln2},$$

**Figure 6:**
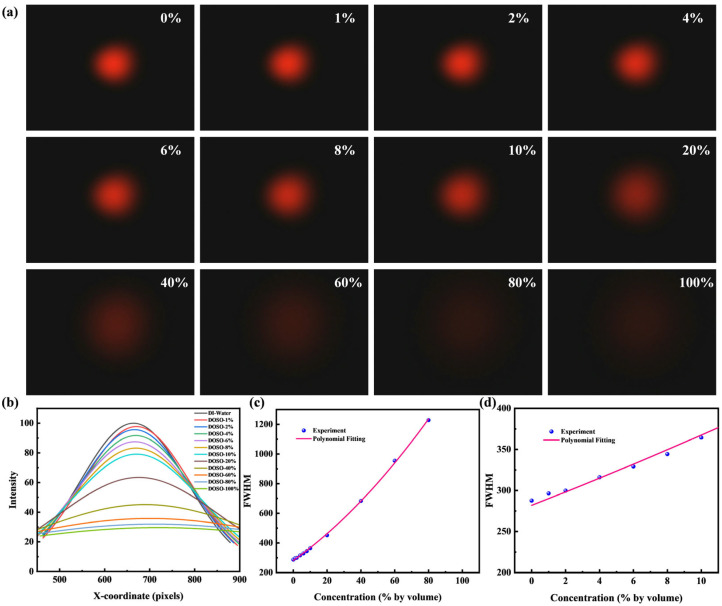
(**a**) Spot images for different DMSO concentrations. (**b**) 1D light intensity distributions for different concentrations. (**c**) Relationship between FWHM and concentration. (**d**) Magnified view at low concentrations.

Figure 6a shows the beam spot images obtained from measuring aqueous solutions of Dimethyl sulfoxide (DMSO), which were prepared with deionized water. The samples cover a concentration range from 0% to 100%, corresponding to a refractive index that varies from 1.333 to 1.483. Initially, we analyzed these inherently 2D images using a conventional one-dimensional (1D) Gaussian fitting approach. This method holds practical value as it represents a traditional analysis technique that is computationally efficient and provides an intuitive, rapid assessment of beam characteristics like intensity and size from a single cross-section, making it suitable for performance-limited situations. Figure 6b presents the intensity distribution profiles from this 1D analysis, while Figure 6c,d illustrate the calculated FWHM. It should be noted that the overall response in Figure 6c is non-linear, especially at higher concentrations. This can be attributed to a combination of the potentially non-linear relationship between refractive index and high solute concentrations, and the inherent non-linear optical response caused by the aspherical geometry of the microlens. However, while the FWHM increases with concentration, the linearity is poor at lower concentrations. We attribute this deficiency to the fact that the 1D method utilizes only a single line of pixels from the entire image, leading to insufficient data utilization and reduced detection accuracy. Therefore, to overcome this limitation, we propose a two-dimensional (2D) Gaussian fitting approach to enhance data utilization.

### 4.2. Fit the Half-Height Area

**Figure 7:**
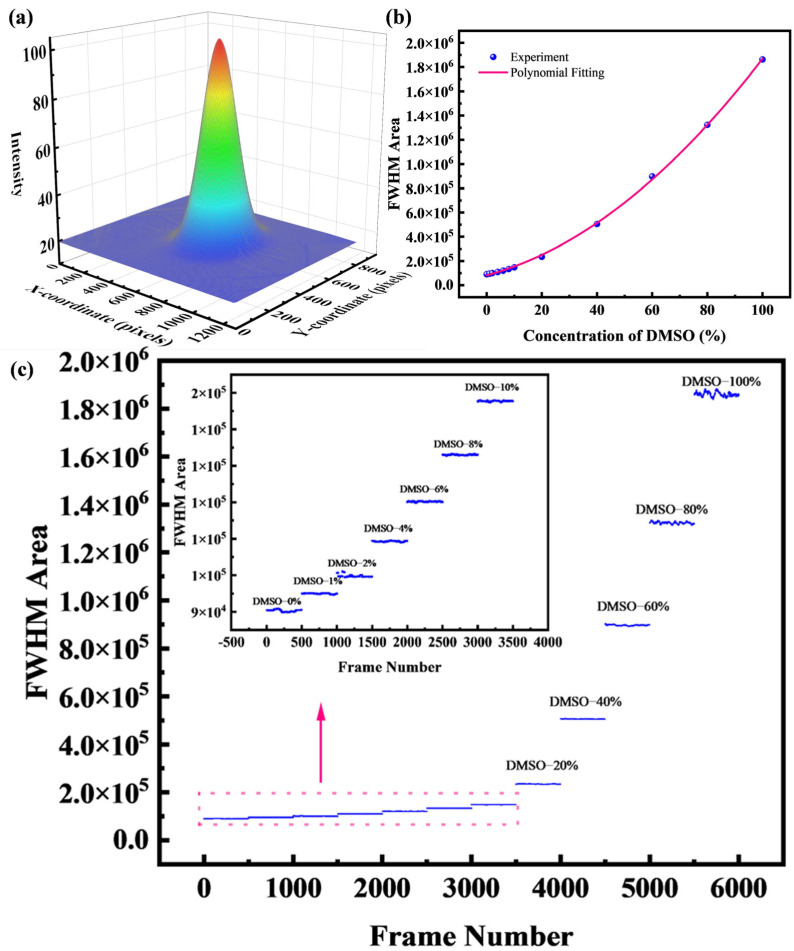
(**a**) Result after 2D Gaussian fitting; (**b**) Relationship between half-height area and concentration; (**c**) Continuous measurements of half-height area, with the upper-left inset (enclosed by a pink dashed box) showing a magnified view.

The 2D Gaussian fitting is performed by converting the original image to grayscale and then carrying out the fitting operation, resulting in a half-maximum area analogous to the full width at half maximum, as shown in Figure 7a. For an ideal Gaussian spot, the light intensity distribution should satisfy the formula.

(7) $$f(x,y)=A\cdot exp\left(-\frac{(x-x_{0}{)}^{2}+(y-y_{0}{)}^{2}}{2\sigma^{2}}\right),$$

However, in practice, an elliptical light spot can be caused by both issues with the laser itself and the asymmetry of microbubbles that results from the fabrication process. Therefore, we introduced corrected Gaussian functions incorporating $\sigma_{x}$, $\sigma_{y}$, and ρ [31]:

(8) $$f(x,y)=A\frac{1}{2\pi \sigma_{x}\sigma_{y}\sqrt{1-\rho^{2}}}exp\left(-\frac{1}{2(1-\rho^{2})}\left[\frac{(x-x_{0}{)}^{2}}{\sigma_{x}^{2}}+\frac{(y-y_{0}{)}^{2}}{\sigma_{y}^{2}}-\frac{2\rho (x-x_{0})(y-y_{0})}{\sigma_{x}\sigma_{y}}\right]\right),$$

$x_{0}$ and $y_{0}$ represent the center of the light spot, $\sigma_{x}$ and $\sigma_{y}$ represent the concentration of the light spot, which is the spot size, ρ represents the correlation coefficient between x and y, used to describe the tilt of the light spot (potentially arising from the asymmetry of the microbubble), and *A* represents the peak light intensity. This two-dimensional Gaussian fitting can maximize the utilization of data to achieve better accuracy. Through the formula:

(9) $$\mathrm{FWHM}\;Area=2\pi \sigma_{x}\sigma_{y}\sqrt{1-\rho^{2}},$$

The desired FWHM area can thus be obtained. Figure 7a is a schematic diagram of the two-dimensional fitting, Figure 7b shows the fitting result of the spot images for 0–100% DMSO concentration. As the DMSO concentration increases from 0% to 100%, the FWHM area exhibits a clear correlation with the concentration, showing a consistent trend of increase.

The above result is based on fitting a single image. If this calculation method is extended to process each frame of a video, it enables continuous frame-by-frame measurements. The video is captured using a CCD with a resolution of 1280 × 960 at 30 frames per second, to minimize random image noise, an average image is generated from every 30 consecutive frames. The resulting denoised image is then subjected to further processing. Based on this approach, measurements were performed for DMSO solutions with concentrations ranging from 0% to 100%. Figure 7c shows the variation of the FWHM area over time, along with a magnified view of the low-concentration region.

**Figure 8:**
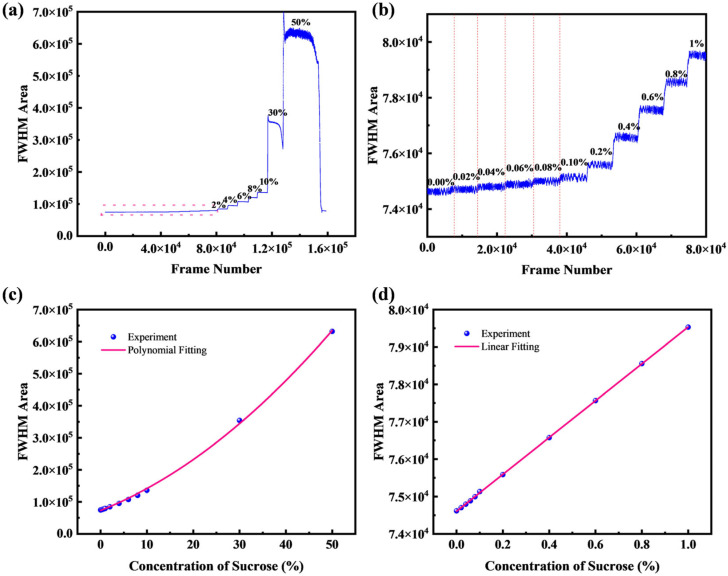
(**a**,**b**) Half-height area of sucrose solutions at different concentrations and magnified view of low concentrations; (**c**,**d**) Variation of half-height area with sucrose concentration and enlarged local view.

Next, continuous measurements were performed on sucrose solutions prepared with deionized water. For these aqueous solutions, the mass concentration was varied from 0% to 50% (refractive index range: 1.333–1.420). The results are shown in Figure 8a,b, with the relationship between concentration and FWHM area plotted in Figure 8c,d; both include magnified views of the 0–1% range. The system demonstrates excellent linearity even at small concentration gradients, achieving a detection limit for sucrose concentration changes of 0.02%, which translates to a refractive index resolution of 3.6 × 10^−5^ RIU. The noise is attributed to an excessive sucrose solution gradient, which results in a non-uniform solution concentration.

**Figure 9:**
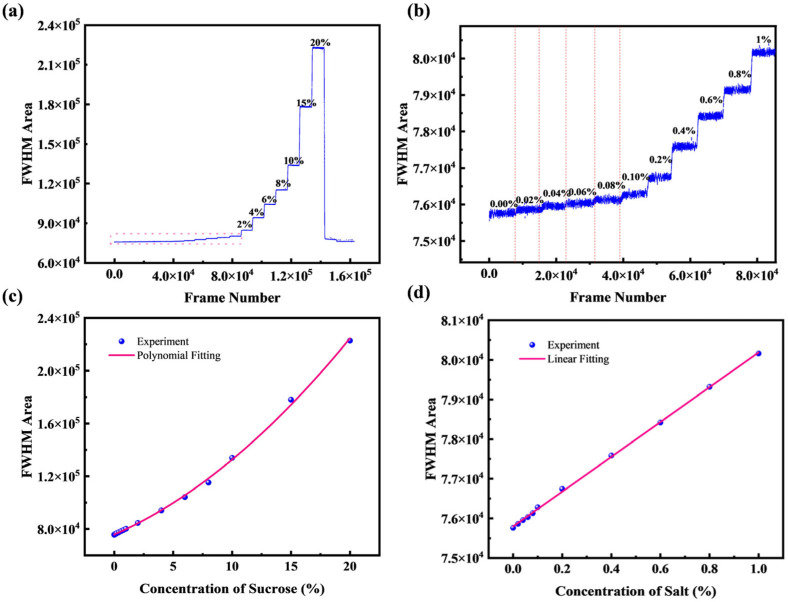
(**a**,**b**) Half-height area of salt solutions at different concentrations and magnified view of low concentrations; (**c**,**d**) Variation of half-height area with salt concentration and enlarged local view.

The system was also tested with salt solutions prepared in deionized water over a mass concentration range of 0% to 20% (refractive index: 1.333–1.368). The continuous measurement results are shown in Figure 9a,b, while the correlation between salt concentration and FWHM area is plotted in Figure 9c,d, both including a magnified view of the 0–1% range. The system achieves a detection limit for salt concentration changes of 0.02%, which translates to a refractive index resolution of 3.6 × 10^−5^ RIU.

The pronounced non-linearity of the FWHM area response at higher concentrations (Figure 8c and Figure 9c) is an inherent characteristic of the measurement principle. While the FWHM itself is largely linear with concentration, the FWHM area is proportional to its square, resulting in a fundamentally quadratic response. The apparent linearity observed in the low-concentration regimes (Figure 8d and Figure 9d) represents the linear portion of this curve, where the first-order term of a Taylor expansion provides an excellent approximation. This allows the system to function as a high-resolution linear sensor for small concentration changes, even though its global response is non-linear.

### 4.3. Gradient Boosting Decision Tree Algorithm

The refractive index can be derived using the Gaussian FWHM area formula. For applications with lower precision requirements, the calculation can be performed using a single image. However, for low concentration gradients, as shown in Figure 8b,d, it is necessary to average the data to obtain more accurate values, which requires a longer measurement time. In contrast, when using the Gaussian fitting formula.

**Figure 10:**
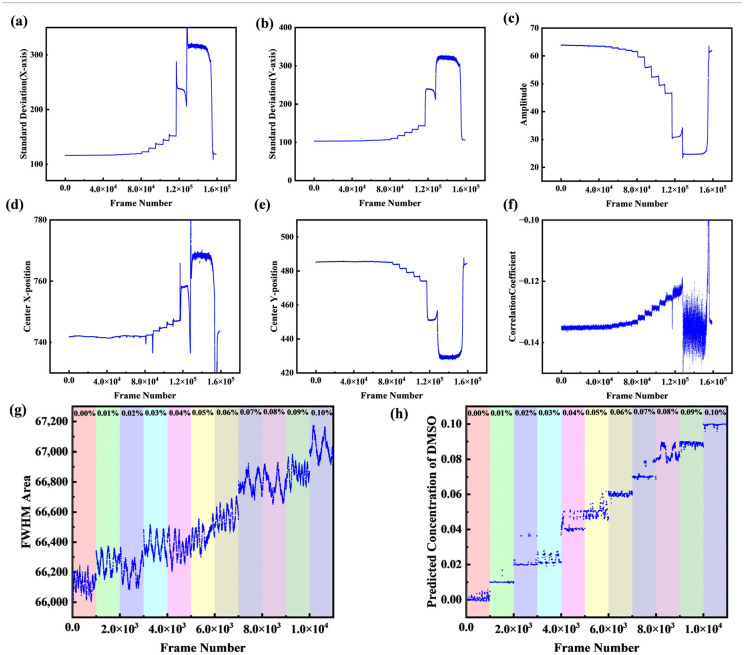
(**a**–**f**) Variation of different parameters with concentration; (**g**) Original data before training; (**h**) Concentration prediction after training.

The calculation of the FWHM area only uses three parameters—$\sigma_{x}$, $\sigma_{y}$, and ρ. However, the actual Gaussian fitting outputs six parameters. To illustrate this, Figure 10a–f show the parameter variations using the data from the previously described sucrose solution experiments. As can be seen, each of these parameters varies with the concentration (although the abrupt changes in subplots (a–e) are caused by a distorted beam spot from incomplete liquid mixing, and the correlation coefficient in subplot (f) proved to be an unreliable indicator). Therefore, we introduce the gradient boosting decision tree algorithm, XGBoost, to learn the complex mapping between concentration and the full set of Gaussian fitting parameters. Moreover, since different parameters exhibit varying sensitivities to noise, this algorithm can also help suppress noise more effectively, thereby enhancing the detection limit.

The model structure used in this study is as follows: the number of trees is set to 100, the maximum depth of each tree is 3, and the learning rate is 0.1. To generate the dataset, all measurements were conducted on a single chip within a single day to ensure consistent experimental conditions. It is important to note that this training was conducted on a relatively small dataset of 11 data groups with the primary goal of demonstrating the feasibility of our method, rather than developing a fully optimized model. The model was trained on aqueous DMSO solutions ranging from 0% to 0.1% with a concentration gradient of 0.01%, resulting in 11 data groups. For each concentration level, approximately 8000 data points (each consisting of the six Gaussian parameters) were collected over time. The dataset was then partitioned using a time-series split: the first 80% of the chronologically collected data for each concentration was used as the training set, while the subsequent 20% was reserved as the test set. To specifically assess the model’s generalization performance on unseen concentrations, the entire datasets for the 0.03% and 0.05% samples were excluded from the training process and used exclusively for testing.

Figure 10g shows the original FWHM area data before training, while Figure 10f presents the predicted concentrations after training. With this prediction method, the measurement time can be shortened to 1 s for low concentration gradients, and the concentration of unknown liquids can be predicted with high accuracy: 98.7% of predictions fall within a ±0.01% error margin (corresponding to 1.4 × 10^−5^ RIU), and 100% of predictions are within a ±0.02% error margin. It is important to note that this calibration is specific to the individual chip and optical alignment. Consequently, the model must be recalibrated for each new device. However, this process is computationally efficient, with training completed in under 10 s on a standard CPU (AMD Ryzen 7 9700X), making it practical for device-specific calibration.

### 4.4. Resolution Affects Discussion

**Figure 11:**
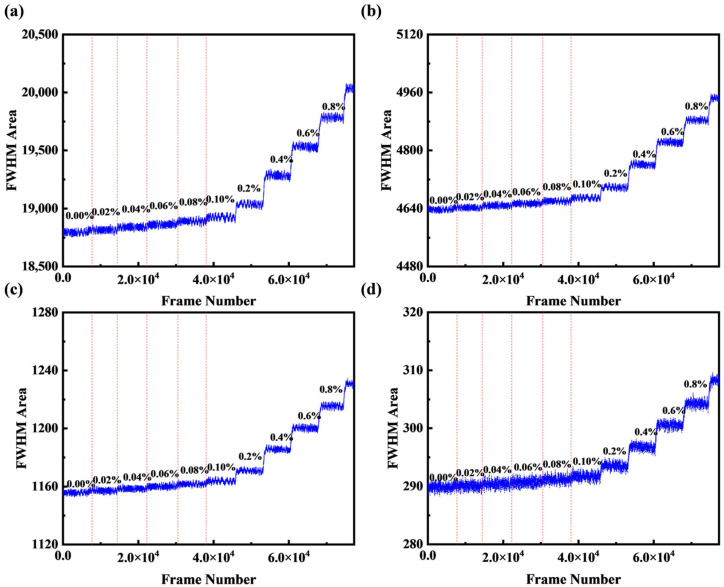
Calculation of FWHM area at different resolutions: (**a**) 640 × 480; (**b**) 320 × 240; (**c**) 160 × 120; (**d**) 80 × 60.

The CCD used in this study has a native resolution of 1280 × 960. To evaluate the impact of resolution, the images were programmatically downsampled by averaging 2 × 2-pixel blocks to create successively lower resolutions: 640 × 480, 320 × 240, 160 × 120, and 80 × 60. Sucrose solutions with concentrations ranging from 0% to 0.8% were processed under these resolutions, as shown in Figure 11a–d. When the resolution decreased from 1280 × 960 to 160 × 120, the noise level did not change significantly; however, upon further reduction to 80 × 60, the noise increased noticeably. This indicates that our fitting-based detection method, which relies on the overall trend across all pixels, does not require high resolution and has the potential for ultra-low-resolution applications.

## 5. Summary

This paper proposes an optofluidic lens-based refractometer that utilizes an extremely simple structure, offering a low-cost and robust solution for refractive index sensing. The device achieves a refractive index detection limit of 1.4 × 10^−5^ RIU and a detection range of 1.33–1.48, which has been experimentally validated using pure saline and sucrose solutions. Its high robustness against structural variations and its lack of stringent requirements for the light source or CCD make it a promising candidate for broad application, particularly for the continuous, real-time monitoring of liquid refractive indices.

This work serves as a successful proof of concept, demonstrating the fundamental feasibility and potential of our method. While the current system and machine learning model are foundational, they provide a strong basis for future enhancements. Future work will proceed in several key directions: First, to bridge the gap between laboratory validation and practical environmental applications, we will test the sensor’s performance with actual water samples, which includes investigating its resilience to potential interferents like suspended particles that could cause CCD speckle noise. Second, we will focus on the further miniaturization and integration of the device to create a compact, field-deployable system. Finally, we will explore the platform’s versatility by adapting it for other sensing modalities, such as force or pressure sensing, leveraging the unique properties of the optofluidic microbubble.

## Figures and Tables

**Table 1:** Comparison of different optical refractometer technologies.

Sensor Type	Sensing Principle	Key Advantages	Major Limitations
Resonance-Based	Resonant peak shift	Extremely high sensitivity	High cost
			Complex fabrication
Traditional Refraction-Based	Critical angle	Simpler principle	No continuous real-time measurement
	Refraction	High precision (lab models)	
This Work	Algorithm-enhanced beam spot analysis	Low cost, high robustness	Depend on computation
		Real-time measurement	

## Data Availability

The data presented in this study are available on request from the corresponding author.
